# Bevacizumab for the treatment of surgically unresectable cervical cord hemangioblastoma: a case report

**DOI:** 10.1186/1752-1947-6-238

**Published:** 2012-08-10

**Authors:** Ayman I Omar

**Affiliations:** 1 Department of Neurology, Division of Neuro-Oncology, Southern Illinois University School of Medicine, 751 N Rutledge St, Springfield, IL, 62704, USA

## Abstract

**Introduction:**

Hemangioblastomas are highly vascular tumors that can arise within the central nervous system as well as other organ systems within the body. They can arise sporadically or as part of von Hippel-Lindau syndrome. Those arising in critical locations within the central nervous system can be difficult to resect surgically and therefore pose a significant challenge and result in morbidity and even mortality. Hemangioblastomas express high levels of vascular endothelial growth factor that drives angiogenesis and tumor progression. We hypothesized that bevacizumab through its inhibitory effect on vascular endothelial growth factor will result in hemangioblastoma tumor regression as well as a meaningful clinical response.

**Case presentation:**

We present the case of a 51-year-old Caucasian man with surgically unresectable cervical cord hemangioblastoma presenting with progressive weakness leading to quadriparesis. He was treated with bevacizumab and his follow up magnetic resonance imaging scans showed marked tumor regression. After only six cycles of intravenous bevacizumab (10mg/kg every two weeks), he started ambulating after being wheelchair bound. He is currently still receiving treatment almost two years after initiation of bevacizumab.

**Conclusions:**

We have shown for the first time that bevacizumab can result in significant tumor regression and a sustained clinical improvement in a patient with an otherwise unresectable spinal cord hemangioblastoma. This novel approach can be immensely useful for patients with difficult to resect hemangioblastomas or those with multiple lesions such as in von Hippel-Lindau syndrome.

## Introduction

Central nervous system (CNS) hemangioblastomas are highly vascular tumors that commonly arise in the brain and less commonly in the spinal cord [1]. Despite their histologically benign nature, they often result in significant neurological disability based upon their location. These tumors express high levels of vascular endothelial growth factor (VEGF) which drives angiogenesis, a process that explains their highly vascular nature [2]. Tumors located within accessible locations in the CNS are commonly treated with surgical resection followed by radiation treatment for incompletely resected or recurrent lesions [3]. A complete resection of these tumors typically results in a cure. However, tumors located in critical locations are occasionally unresectable and, therefore, cures are rarely achieved with non-operative management such as radiation therapy [4]. Furthermore, radiation carries the risk of increasing peritumoral edema as well as the potential for development of radiation necrosis [5]. To date, chemotherapy and/or targeted agents play a limited role in this patient population [6].

Given their high levels of VEGF expression, blocking the VEGF pathway would be an appealing target for controlling angiogenesis and subsequently tumor growth. Bevacizumab is a humanized monoclonal antibody that binds to the VEGF protein and inhibits its interaction with the VEGF receptor [7]. It is, therefore, a logical candidate for the treatment of surgically unresectable hemangioblastomas. This targeted agent was recently approved by the US Food and Drug Administration (FDA) for the treatment of recurrent glioblastoma, but to the best of our knowledge, there are no published reports of spinal cord hemangioblastoma regression following administration of this agent. Anti-angiogenic agents such as thalidomide have been tested in the past with stabilization of spinal cord hemangioblastoma in a pediatric patient with von Hippel-Lindau syndrome (VHL) [8]. In addition, a more recent report indicates that retinal hemangioblastomas may respond favorably to bevacizumab [9]. Another case report showed that treatment with intravitreal bevacizumab had no effect on a hemangioblastoma of the optic nerve head [10]. To the best of our knowledge, our report is the first to show tumor regression as well as sustained and meaningful clinical improvement in a patient with unresectable spinal cord hemangioblastoma treated with bevacizumab.

## Case presentation

A 51-year-old Caucasian man with a four-year history of left hand pain, dysesthesia and weakness that progressed over the subsequent year to involve the left upper extremity presented to our clinic. He was initially diagnosed with Raynaud’s disease and treated with nifedipine. His pain continued to worsen and later progressed to involve bilateral upper extremities all the way up to the elbows. Over the subsequent two to three years he developed progressive right upper extremity as well as bilateral lower extremity weakness. Neurologic examination revealed quadriparesis and impaired sensation to all modalities over bilateral upper extremities up to the elbows and bilateral lower extremities up to a few inches below the waistline.

He underwent a magnetic resonance imaging (MRI) of the brain and cervical spine which showed a 2.7 × 1.1 × 1.5cm avidly enhancing intramedullary lesion at C2-C4 with a cervical cord syrinx, marked cord edema and expansion, extending from the medulla through T3. He was taken to the operating room and an attempt at surgical resection was unsuccessful due to the lack of a defined surgical plane and the high vascularity of the tumor mass. A biopsy was performed which confirmed the diagnosis of hemangioblastoma. The syrinx was not entered or drained during this procedure. The patient refused testing for von Hippel-Lindau disease but no other hemangioblastomas were seen on whole neuraxis scanning and fundoscopic examination. Postoperatively he continued to deteriorate to the extent that he became wheelchair bound. Oral dexamethasone (16mg daily) was initiated approximately one week postoperatively because of increasing arm weakness and he showed minimal improvement. The improvement was short-lived and dexamethasone was subsequently tapered over the ensuing two to three weeks.

**Figure 1:**
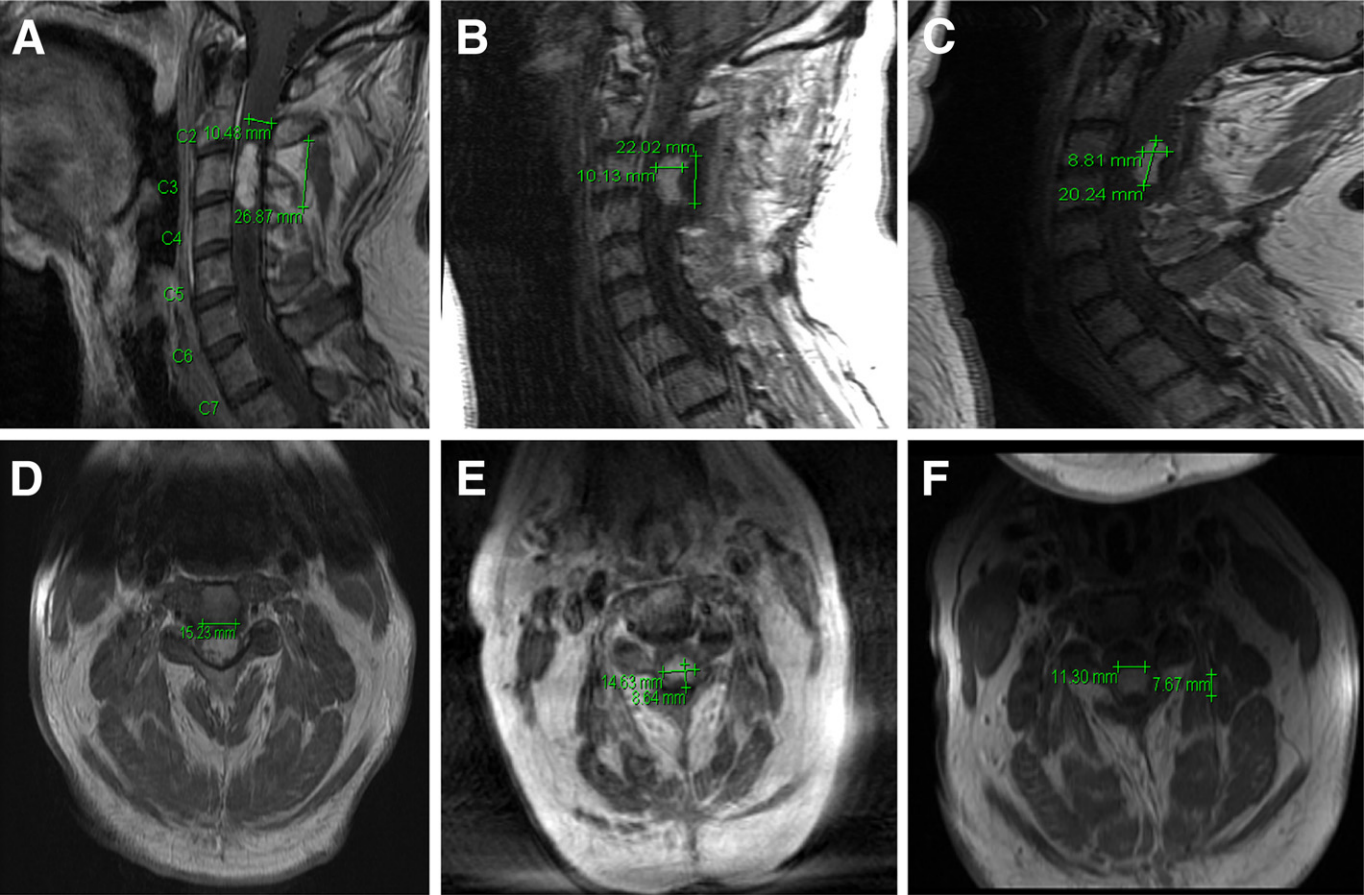
**Bevacizumab resulted in tumor regression of a cervical cord hemangioblastoma.** This is a sagittal and axial magnetic resonance imaging view of the cervical spine, post gadolinium-diethylene triamine pentaacetic acid administration. Figure 1**A** (sagittal) and 1**D** (axial) demonstrate the pre-bevacizumab baseline scan. Figure 1**B** (sagittal) and 1**E** (axial) demonstrate tumor regression following two cycles of bevacizumab. Figure 1**C** (sagittal) and 1**F** (axial) demonstrate further reduction in the enhancing tumor following an additional four cycles of bevacizumab.

Bevacizumab was started after obtaining appropriate informed consent. The patient was given a 10mg/kg infusion every two weeks (initially for two cycles) and an MRI of the cervical spine was obtained. The MRI revealed significant improvement in the T2 signal change surrounding the enhancing component and a decrease in the size of enhancing disease from 2.7 × 1.0 × 1.5cm to 2.2 × 1.0 × 1.5cm (Figure 1A, 1B, 1D and 1E). His arm strength improved from 3/5 to 4/5 bilaterally but he was receiving rehabilitation treatment during that time. He remained wheelchair bound with 2/5 strength in both lower extremities. Four additional cycles of bevacizumab were given (to a total of six cycles) and a repeat MRI showed a further decrease in tumor dimensions, now measuring 2.0 × 0.8 × 1.5cm (Figure 1C and 1F). He subsequently developed arthralgia and elected to stop bevacizumab treatment but he had already started taking a few steps with assistance. Bevacizumab was restarted after several months and he showed slow clinical improvement. Currently, he is able to ambulate about 200 feet with minimal assistance after two years of bevacizumab treatment.

## Discussion

Hemangioblastomas are benign highly vascular central nervous system tumors. They represent 1.5% to 2.5% of all intracranial tumors, the majority of which are located within the cerebellar hemispheres [1]. Other CNS locations are less common and include the cerebral hemispheres (9%), brainstem (5%), and spinal cord (7%) [1]. They may occur as part of the VHL complex in up to 25% of cases or more commonly as sporadic lesions in up to approximately 75% of cases [11].

The molecular pathogenesis of hemangioblastomas is not fully understood; however, in VHL disease the genetic mutation had been previously elucidated. The disease is linked to a mutation of the *VHL* gene on chromosome 3p25-26 which transcribes VHL protein (pVHL) [12]. This protein is normally responsible for degradation of the hypoxia-inducible factor (HIF) transcriptional complex. Under hypoxic conditions, HIF upregulates expression of growth factors, such as vascular endothelial growth factor (VEGF), platelet-derived growth factor (PDGF), and erythropoietin. Loss of *VHL* function results in deregulated HIF activity and overexpression of VEGF leading to hemangioblastomas [11]. Since VEGF and its receptors appear to play a crucial role in hemangioblastoma tumorigenesis, we hypothesized that blocking the VEGF pathway might result in disruption of tumor angiogenesis and consequently tumor regression. In this report, we demonstrate for the first time the successful treatment of a surgically unresectable cervical cord hemangioblastoma using bevacizumab (a monoclonal antibody directed against VEGF) [7]. Bevacizumab is currently used for the treatment of a variety of solid tumors including recurrent glioblastomas, metastatic colorectal carcinoma, and breast cancer as well as renal cell carcinoma [7]. Intravitreal bevacizumab was attempted in previous series for the treatment of retinal hemangioblastomas with varying degrees of success [9,10].

Current treatment options for patients with CNS hemangioblastomas include surgical resection as well as adjuvant stereotactic radiosurgery for incompletely resected or recurrent lesions [13,14]. Although gross total resection commonly results in excellent outcomes, in some circumstances given the highly vascular nature of these tumors, a gross total resection is not feasible. For example, in one series a gross total resection was achieved in only three of six patients (50%) with intramedullary spinal cord hemangioblastomas [4]. In these instances, radiosurgery is commonly employed for incompletely resected lesions. However, the therapeutic efficacy of radiosurgery diminishes over time and post-radiation swelling can result in significant morbidity and neurologic decline [14]. In addition, radiation necrosis has been reported following radiosurgical treatment, a complication which can result in poor outcomes for those patients [5].

Development of a systemic approach would, therefore, offer an attractive alternative for patients with surgically unresectable hemangioblastomas, those with medical comorbidities that may preclude surgery as well as patients with multiple hemangioblastomas as can be seen with the VHL complex. We have shown that the systemic infusion of bevacizumab can result in hemangioblastoma tumor regression as well as a sustained and meaningful clinical improvement. This effect may potentially be attributed to a decrease in vascular permeability and subsequent improvement of edema, regression of angiogenesis or a combination of both. Further studies are needed to define better the role of bevacizumab in the treatment of this challenging disease.

## Conclusions

Only one case report was found in a Medline search (keywords: bevacizumab; hemangioblastoma) that showed retinal hemangioblastomas may respond favorably to intravitreal bevacizumab [9]. To the best of our knowledge, this is the first report to demonstrate that bevacizumab results in a positive radiographic response as well as significant clinical improvement (from wheelchair-bound to ambulating with minimal assistance) when used to treat a surgically unresectable spinal cord hemangioblastoma.

## Consent

Written informed consent was obtained from the patient for publication of this case report and any accompanying images. A copy of the written consent is available for review by the Editor-in-Chief of this journal.

## Abbreviations

CNS: central nervous system; HIF: hypoxia-inducible factor; PDGF: platelet-derived growth factor; pVHL: von Hippel-Lindau protein; US FDA: United States Food and Drug Administration; VEGF: vascular endothelial growth factor; VHL: von Hippel-Lindau.

## Competing interests

The author declares that he has no competing interests*.*
